# Pediatric ileoileal intussusception with a lipoma lead point: a case report

**DOI:** 10.1093/gastro/got032

**Published:** 2013-12-19

**Authors:** Yoshihide Asaumi, Tamon Miyanaga, Yasuhiro Ishiyama, Masakazu Hattori, Yasuo Hashizume

**Affiliations:** Department of Surgery, Fukui Prefectural Hospital, Fukui, Japan

**Keywords:** Lipoma, Intussusception, Child

## Abstract

Intussusception is a common cause of mechanical bowel obstruction among children, with older children being more likely to have a pathological lead point. Intestinal neoplasms are rare and small intestinal lipomas are even less common. Herein we describe a case of a 7-year-old boy with ileoileal intussusception, with an ileal lipoma as the pathological lead point. Computed tomography was useful pre-operatively for revealing intussusception due to lipoma as the pathologic lead point.

## INTRODUCTION

Intussusception in a child during the first year of life is sometimes caused by ileal hyperplasia. Lipoma of the ileum is a rare tumor type, accounting for only approximately 5% of all gastrointestinal tumors in adults. We describe an ileoileal intussusception due to lipoma in a 7-year-old child.

## CASE REPORT

**Figure 1.**
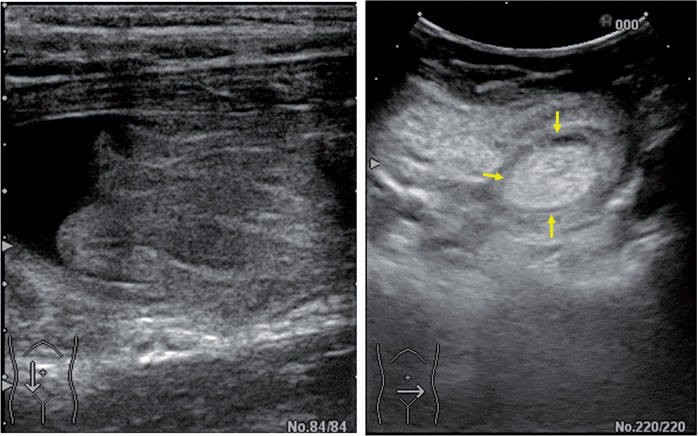
Ultrasonography with transverse view showing echogenic mass, which has pseudokidney appearance.

**Figure 2.**
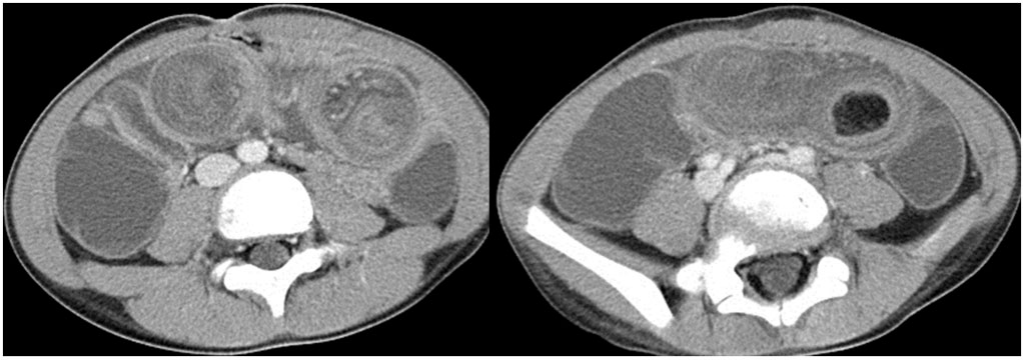
Ileoileac intussusception, fat density mass suspected lipoma was discovered to be the pathologic lead point.

A 7-year-old boy with a 3-day history of abdominal pain was treated with medication at another hospital; his past medical history and family history were unremarkable. Because his symptoms continued to worsen, he was brought to our hospital. An ultrasonographic examination revealed intussusception (Figure 1) and enema was unsuccessfully attempted to reduce the intussusception. Computed tomography (CT) revealed an ileoileal intussusception with a fatty dense mass as the pathologic lead point (Figure 2). Thus, the patient was diagnosed with lipoma and intussusception, and surgical reduction was recommended.

**Figure 3.**
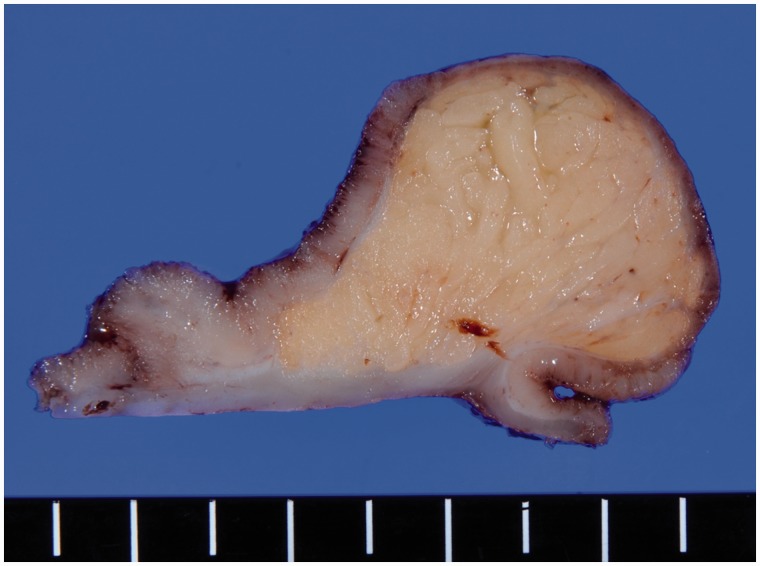
Specimen of resected ileum revealing submucosal tumor as a lead point of intussusception.

An exploratory open laparotomy was performed and a palpable tumor was detected in the ileum, 50 cm from the ileocolic valve on the oral side. A wedge resection of the ileum resulted in complete resection of the tumor (Figure 3). The patient’s post-operative recovery was uncomplicated, and he was discharged 8 days after the operation. A pathologic examination confirmed the tumor as a lipoma of the ileum.

## DISCUSSION

Intussusceptions are occasionally observed in children, with an incidence of approximately 50 per 100 000 individuals [1, 2]; this incidence is lower among children younger than 3 months or older than 6 years [3]. Among children, idiopathic intussusception is most common, with fewer than 5% of cases being associated with a tumor [4]. Intussusceptions occurring in older children are more likely to have a pathologic lead point, such as an inverted Meckel diverticulum, a benign polyp, or a lymphoma [5].

Gastrointestinal lipomas are most commonly located in the colon, with approximately 20–25% of these tumors occurring in the small bowel [6]. Most lipomas are solitary but they can be multiple and located anywhere in the gastrointestinal tract [7]. Lipomas are mainly asymptomatic but, when symptoms do present, these are intestinal obstructions and hemorrhage in most cases [8]. Lipomas usually manifest themselves as highly echoic masses under ultrasonographic examination or as round or oval, well-defined, hypointense, intraluminal masses on CT scans [9].

Among children, ileoileal intussusception due to an ileal lipoma as the pathological lead point is rare. In fact, to our knowledge, this is the first reported case of its kind [10]. In this case, a diagnosis was successfully made using both ultrasonographic- and CT examinations pre-operatively, showing the benefit of diagnostic imaging in the effective diagnosis of intussusception in older children [11].

**Conflict of interest:** none declared.
